# Relationship between MRI T2 mapping parameters and patellar ligament degeneration in patients with knee osteoarthritis

**DOI:** 10.1097/MD.0000000000046501

**Published:** 2026-01-23

**Authors:** Kaifu Wang, Jie Men, Cuixia Mao, Xiaoyi Lao, Zhanqing Zhang, Xing Li, Zuanming Huang, Xiesheng Xu

**Affiliations:** a Guangzhou University of Chinese Medicine Maoming Hospital, Maoming, Guangdong Province, China.

**Keywords:** degeneration, degeneration grading, Kellgren–Lawrence grading, knee osteoarthritis, MRI, patellar ligament, t2 mapping

## Abstract

This study aims to investigate the relationship between MRI T2 mapping parameters and patellar ligament degeneration in patients with knee osteoarthritis (KOA) and evaluate its value in disease grading and prediction of clinical symptoms. A total of 149 KOA patients (284 knees) and 80 healthy subjects (141 knees) who underwent knee MRI between February 2023 and February 2024 were retrospectively analyzed. T2 mapping was performed, and the patellar ligament was divided into proximal, middle, and distal regions. Regions of interest were delineated to measure the mean (T2mean) and maximum (T2max) T2 values. Ligament degeneration was graded on a 0 to 3 scale, and KOA severity was assessed using Kellgren–Lawrence (KL) grading. Group comparisons, correlation and regression analyses, and receiver operating characteristic curve evaluation were performed to determine the diagnostic and predictive value of T2 parameters for degeneration and clinical symptoms (visual analog scale, Western Ontario and McMaster Universities Osteoarthritis Index). T2mean and T2max in all patellar ligament regions were significantly higher in KOA patients than in healthy controls (all *P* < .001). T2 values increased progressively with higher degeneration grades and KL grades (*P* < .001), and even grade 0 KOA cases showed elevated values compared with controls (*P* < .05). T2 parameters were positively correlated with visual analog scale and Western Ontario and McMaster Universities Osteoarthritis Index scores (*r* = 0.37–0.46, all *P* < .001). Multiple regression analysis identified T2mean as an independent predictor of pain and function. Receiver operating characteristic analysis demonstrated that proximal T2mean had the highest diagnostic performance for moderate-to-severe degeneration (area under the curve = 0.82; cutoff = 42.5 ms; sensitivity = 76%; specificity = 75%), with no significant differences among regions (*P* > .05). MRI T2 mapping sensitively detects microstructural degeneration of the patellar ligament in KOA. T2 values rise with increased degeneration and KL grade and correlate significantly with pain and functional limitation. Patellar ligament T2 parameters may serve as quantitative imaging biomarkers for early detection, grading, and clinical assessment of KOA.

## 1. Introduction

Knee osteoarthritis (KOA) is one of the most common chronic degenerative joint diseases worldwide. Its pathological basis includes cartilage degeneration, joint space narrowing, subchondral bone sclerosis, osteophyte formation, and synovial inflammation.^[1,2]^ Epidemiological data show that the incidence of KOA increases significantly with aging and the rise in obesity, particularly in middle-aged and elderly populations. It is a major cause of pain, joint dysfunction, and reduced quality of life.^[3,4]^ Although the Kellgren–Lawrence (KL) grading system on X-ray remains the main clinical basis for assessing KOA severity, it primarily reflects changes in bony structures and cannot sensitively capture early degenerative changes of joint soft tissues, especially the patellar tendon.^[5]^

The patellar tendon (PT), connecting the patella and the tibia, plays a central role in maintaining knee extension function and stability.^[6,7]^ PT degeneration may not only directly cause anterior knee pain but also exacerbate abnormal joint load distribution, thereby promoting the progression of KOA.^[8]^ Previous studies suggest that KOA patients often present with degenerative changes in the PT, including collagen fiber disorganization, increased water content, abnormal signal intensity, and thickening.^[9]^ These pathological changes are often closely related to the severity of pain and functional impairment, indicating that the PT may play an important role in the pathogenesis of KOA.^[10]^ However, accurate and quantitative imaging-based evaluation remains a clinical challenge.

Conventional magnetic resonance imaging (MRI) has certain advantages in evaluating morphological changes of the PT, but routine sequences are mostly qualitative and cannot reflect early microstructural damage.^[10]^ In recent years, functional MRI techniques have been increasingly applied in musculoskeletal disease research. Among these, T2 mapping, which quantitatively reflects changes in collagen fiber orientation and water content, has shown good value in assessing degenerative changes of cartilage, meniscus, and other structures.^[11]^ Prolonged T2 values often indicate matrix damage and increased water content, making them a sensitive marker of early degeneration.^[11]^ However, research on the application of T2 mapping in PT degeneration is still limited, and its relationship with KOA clinical symptoms and pathological grading has not been systematically clarified.

A few studies suggest that PT T2 values are significantly elevated in KOA patients and positively correlated with clinical symptoms. Nonetheless, several unresolved issues remain: first, along the longitudinal axis, the PT can be divided into proximal, middle, and distal regions, but whether degenerative changes differ among these regions is unclear^[12]^; second, the dynamic variation of T2 values in relation to KOA imaging grading (e.g., KL grading) and PT degeneration grading requires further validation^[12]^; third, whether T2 mapping parameters can independently predict the severity of clinical symptoms needs to be examined using multivariate regression models.^[12]^ Moreover, a systematic evaluation of the diagnostic performance of T2 values for moderate-to-severe PT degeneration is still lacking.

Based on this, the present study retrospectively collected large-sample data of KOA patients and healthy controls. MRI T2 mapping was applied for quantitative zonal analysis of the PT. By comparing T2 parameters across different regions, degeneration grades, and KL grades, the study explored their correlation with KOA clinical symptoms (visual analog scale [VAS], Western Ontario and McMaster Universities Osteoarthritis Index [WOMAC]) and further evaluated their independent predictive value using regression models. Additionally, receiver operating characteristic (ROC) curve analysis was employed to verify the diagnostic performance of PT T2 values for moderate-to-severe degeneration. This study aims to reveal the potential value of PT T2 mapping in imaging assessment and clinical prediction of KOA, providing a new quantitative imaging basis for early diagnosis and graded management of KOA.

## 2. Materials and methods

### 2.1. Study subjects

This study was approved by the Ethics Committee of Guangzhou University of Chinese Medicine Maoming Hospital. A total of 149 patients with knee osteoarthritis who visited our hospital between February 2023 and February 2024 were collected, involving 284 knees. Additionally, 80 healthy subjects from physical examinations during the same period, involving 141 knees, were selected as the control group.

#### 2.1.1. Inclusion criteria for the healthy control group

Subjects undergoing health examinations or voluntarily participating in imaging examinations at our hospital during the same period; aged ≥ 40 years; generally matched to the study group in terms of sex and age distribution; no clinical symptoms such as knee pain, swelling, or stiffness; no history of knee trauma or surgery; no history of rheumatoid arthritis, ankylosing spondylitis, gout, or other joint-related diseases; knee MRI showing no significant abnormalities, with good quality T2 mapping images suitable for analysis; complete clinical data; and voluntary signing of informed consent.

#### 2.1.2. Inclusion and exclusion criteria for the arthritis group

##### 2.1.2.1. Inclusion criteria

Meeting the diagnostic criteria for KOA established by the American College of Rheumatology; aged ≥ 40 years; knee MRI including complete T2 mapping sequences with good image quality suitable for analysis; and complete clinical and imaging data.

##### 2.1.2.2. Exclusion criteria

History of severe knee trauma or surgery (e.g., anterior cruciate ligament reconstruction, extensive arthroscopic debridement, etc.); presence of other joint diseases such as rheumatoid arthritis, ankylosing spondylitis, or gouty arthritis; acute patellar tendon injury, tear, or infectious lesion; presence of metallic implants or severe motion artifacts affecting MRI image quality; and severe systemic diseases (such as heart, liver, or kidney failure) making examination or follow-up impossible.

### 2.2. Data collection

#### 2.2.1. Clinical data

Extracted from the electronic medical record system: age, sex, height, weight, and body mass index (BMI); side of the affected knee; duration of symptoms; medical history (smoking, diabetes, etc.); physical activity/exercise level (graded or questionnaire-based); pain and functional scores (WOMAC, VAS, etc.); and X-ray Kellgren–Lawrence (KL) grading (0–IV, independently assessed by 2 musculoskeletal radiologists, with discrepancies resolved through discussion).

#### 2.2.2. MRI examination and T2 mapping sequence

**Figure 1.**
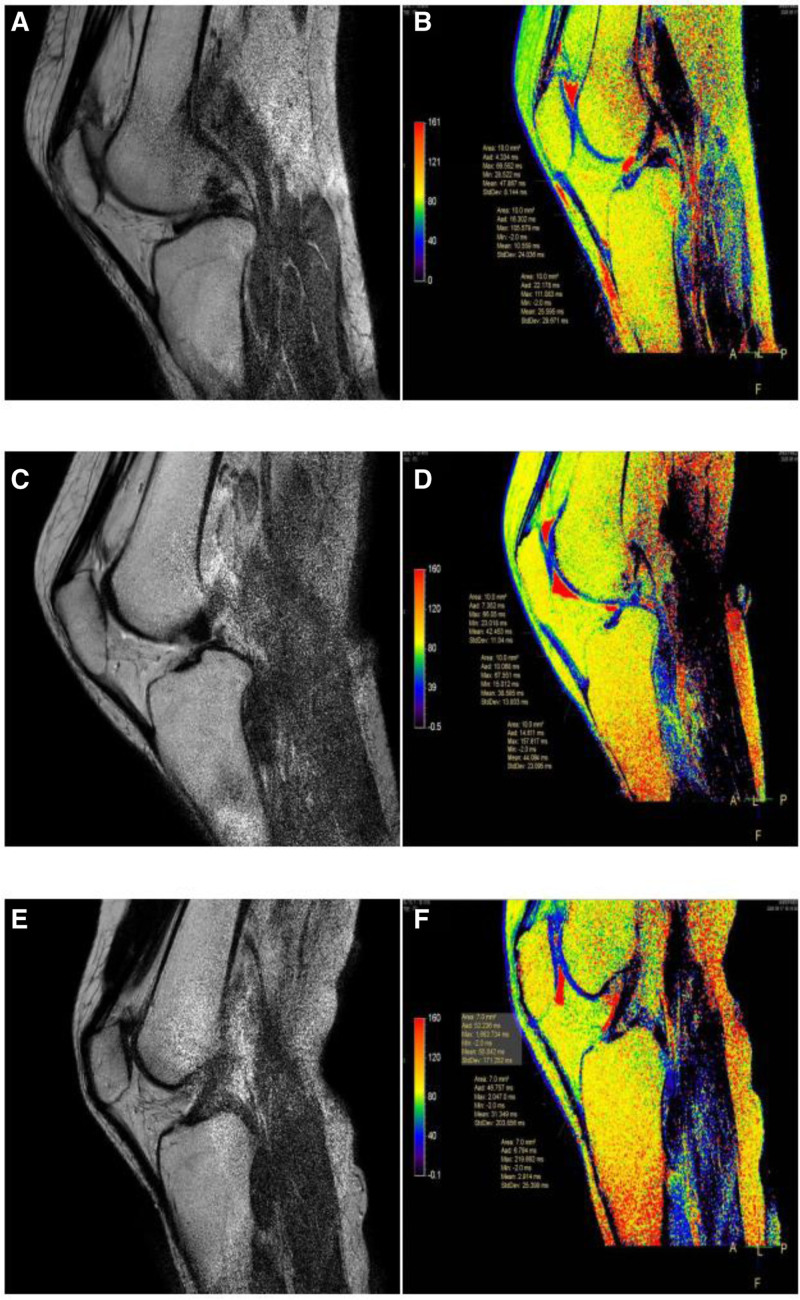
A, C, E represent the original images of T2 mapping; B, D, F are the pseudo-color images of T2 mapping on the same layer.

An Ingenia 3.0T MRI scanner (Philips, Netherlands) was used. All subjects were examined with an 8-channel phased-array knee–ankle joint coil in the supine position, feet first, with the knee naturally extended and the inferior margin of the patella positioned at the center of the coil. The scanning sequences and parameters are shown in Table 1. An example of a T2 mapping image is shown in Figure 1.

**Table 1:** Baseline demographic and clinical characteristics.

Variable	KOA group (n = 149, knees = 284)	Control group (n = 80, knees = 141)	Statistic (t/χ²)	*P* value
Age (yr)	64.7 ± 7.8	63.9 ± 7.5	0.71	.478
Sex (male/female)	56/93	29/51	0.04	.842
BMI (kg/m²)	26.1 ± 3.4	25.7 ± 3.1	0.87	.386
Examined side (unilateral/bilateral)	14/135	19/61	1.42	.233
Knee side (left/right)	141/143	70/71	0.01	.922
Smoking history	22 (14.8%)	10 (12.5%)	0.2	.655
Alcohol consumption	28 (18.8%)	13 (16.3%)	0.19	.667
Diabetes mellitus	20 (13.4%)	9 (11.3%)	0.21	.647
Hypertension	43 (28.9%)	20 (25.0%)	0.39	.534
VAS score (0–10)	5.8 ± 1.9	0.7 ± 0.6	24.6	<.001
WOMAC total score (0–96)	45.2 ± 15.7	5.8 ± 3.4	22.8	<.001
Symptom duration (yr)	6.2 ± 3.9	–	–	–
<5 yr, n (%)	68 (45.6%)	–	–	–
≥5 yr, n (%)	81 (54.4%)	–	–	–

BMI = body mass index, KOA = knee osteoarthritis, VAS = visual analog scale, WOMAC = Western Ontario and McMaster Universities Osteoarthritis Index.

#### 2.2.3. ROI delineation and quantitative analysis

Post-processing of T2 mapping images was performed directly on the Philips MR workstation using exponential fitting. The pseudocolor threshold was uniformly set from 0 ms (blue) to 160 ms (red) to obtain a standardized distribution range of T2 values, facilitating comparisons among different subjects.

##### 2.2.3.1. Patellar tendon segmentation method

According to anatomical landmarks, the patellar tendon (PT) was divided along its longitudinal axis into 3 regions: proximal, middle, and distal. The proximal patellar tendon was defined as the region from the inferior pole of the patella to the proximal one-third of the tendon length; the middle patellar tendon as the region from one-third to two-thirds of the tendon length; and the distal patellar tendon as the region from the distal third of the tendon length to the tibial tuberosity. On the optimal plane perpendicular to the tendon axis, 2 to 3 consecutive slices were selected for analysis to ensure representativeness of sampling.

##### 2.2.3.2. ROI delineation principles

All region of interest (ROIs) were independently delineated in a blinded manner by 2 radiologists with ≥ 5 years of experience in musculoskeletal imaging. ROIs were placed in the central portion of the PT, avoiding marginal areas, calcifications, vessels, and obvious artifacts, to ensure uniform and reliable signal measurements. When necessary, registration with corresponding proton density fat-saturated anatomical sequences was performed to ensure accurate and consistent ROI positioning. Within each region, 3 small ROIs of approximately 1 mm² were selected, and their mean value was taken as the quantitative result for that region.

##### 2.2.3.3. Quantitative parameters

This study recorded and analyzed multiple quantitative parameters for each PT region, including mean T2 value (T2mean), maximum T2 value (T2max), and T2 histogram percentiles (e.g., P10, P50, P90, optional). T2mean was used to reflect overall changes in collagen matrix and water content in the region, while T2max was helpful in capturing focal degenerative signals.

##### 2.2.3.4. Quality control

For quality control, any slice with significant fitting residuals, insufficient echo numbers, or obvious motion or metal artifacts was excluded. If fewer than 2 effective slices of the PT in a given knee were available for analysis, that knee was excluded from the quantitative assessment. In addition, to further evaluate measurement reliability, 2 radiologists underwent unified training and performed the initial delineations; one of them repeated the measurements independently after 2 weeks, and the results were used to assess intra- and inter-observer consistency.

#### 2.2.4. Evaluation of patellar tendon degeneration and KOA severity

Patellar tendon degeneration was graded on MRI using a custom 0 to 3 scale for statistical analysis. Grade 0 indicated homogeneous signal and normal thickness; Grade 1 indicated focal or diffuse mild T2 hyperintensity with mild thickening (thickness increase less than 20% compared with the average tendon thickness); Grade 2 indicated moderate diffuse hyperintensity with moderate thickening (20–50%) or irregular morphology; and Grade 3 indicated marked hyperintensity with significant thickening (greater than 50%), disorganized tendon fibers, or signs of partial tear. Grading was independently performed by 2 readers, and the consensus result was adopted; in case of disagreement, a senior radiologist made the final judgment. The severity of KOA was assessed using X-ray KL grading (0–IV), and the presence of patellofemoral joint degeneration signs such as subchondral sclerosis and osteophytes was also recorded.

Inter-observer agreement and test–retest reliability were evaluated by having 2 readers independently repeat ROI delineation and grading on 30 randomly selected knees after an interval of at least 2 weeks. The intraclass correlation coefficient (ICC, two-way random, consistency/absolute agreement model) was calculated to assess test–retest reliability and inter-observer agreement of T2 quantification, while weighted Kappa was calculated for grading data to evaluate agreement and systematic bias.

### 2.3. Statistical analysis

All statistical analyses were performed using SPSS software (version 26.0; IBM Corp., Armonk). Continuous variables confirmed to follow a normal distribution by the Shapiro–Wilk test were expressed as mean ± standard deviation (x̄ ± s). Non-normally distributed continuous variables were expressed as median and interquartile range. Categorical variables were expressed as frequency and percentage. Group comparisons were conducted using independent-samples *t* tests for continuous variables such as demographic characteristics and T2 values between KOA and control groups, or χ² tests for categorical variables such as sex and medical history. Multiple group comparisons were performed using one-way analysis of variance (ANOVA), with post hoc pairwise comparisons by LSD test, for example, for T2 values among different degeneration grades or KL grades. Linear trend tests were used to analyze changes in T2 values with increasing degeneration or KL grade.

Pearson correlation analysis was applied to evaluate the relationship between patellar tendon T2 values and clinical symptom scores, including VAS and WOMAC. Multiple linear regression analysis was used to explore the effect of patellar tendon T2 values on clinical symptom scores, with adjustment for confounding factors such as age, sex, BMI, and KL grade. ROC curve analysis was performed to evaluate the diagnostic performance of T2 values for moderate-to-severe patellar tendon degeneration (≥grade 2), calculating the area under the curve (AUC), optimal cutoff value, sensitivity, and specificity. The DeLong test was applied to compare differences in AUC of T2 values among different tendon regions. The ICC was used to assess intra- and inter-observer consistency of T2 measurements, while the weighted Kappa coefficient was used to assess the consistency of patellar tendon degeneration grading. ICC values greater than 0.75 or Kappa values greater than 0.6 were considered indicative of good consistency. All tests were two-sided, and *P* < .05 was considered statistically significant.

## 3. Results

### 3.1. Comparison of baseline demographic and clinical characteristics

A total of 149 KOA patients (284 knees) and 80 healthy controls (141 knees) were included. The mean age of the KOA group was 64.7 ± 7.8 years (range 45–79 years), while that of the control group was 63.9 ± 7.5 years, with no statistically significant difference between the 2 groups (see Table 1). There were also no significant differences between the groups in sex ratio, BMI, side of examination, smoking history, drinking history, or prevalence of diabetes and hypertension.

In terms of clinical symptoms, the KOA group had significantly higher VAS scores (5.8 ± 1.9 vs 0.7 ± 0.6) and WOMAC total scores (45.2 ± 15.7 vs 5.8 ± 3.4) compared with the control group (both *P* < .001). The average disease duration in the KOA group was 6.2 ± 3.9 years, of which 45.6% had a disease duration of less than 5 years and 54.4% had a duration of 5 years or more.

### 3.2. Comparison of patellar tendon T2 values between KOA group and healthy controls

Quantitative analysis of MRI T2 mapping showed that the T2 values of all patellar tendon regions in KOA patients were significantly higher than those in the healthy control group (all *P* < .001), Table 2. Specifically, the mean T2 values (T2mean) of the proximal, middle, and distal regions of the patellar tendon in the KOA group were 42.8 ± 5.7, 40.2 ± 5.1, and 41.5 ± 5.4 ms, respectively, while the corresponding values in the control group were significantly lower. Similarly, the T2max, reflecting focal signal changes, were also significantly elevated in all regions of the KOA group compared with controls (proximal: 52.6 ± 6.3 vs 45.1 ± 5.8 ms; middle: 49.7 ± 5.8 vs 42.3 ± 5.2 ms; distal: 50.8 ± 6.0 vs 44.0 ± 5.5 ms).

**Table 2:** Comparison of patellar tendon T2 values between KOA and control groups.

Region of patellar tendon	KOA group (n = 284 knees)	Control group (n = 141 knees)	*t* value	*P* value
Proximal T2mean	42.8 ± 5.7	36.4 ± 4.9	11.2	<.001
Proximal T2max	52.6 ± 6.3	45.1 ± 5.8	10.3	<.001
Middle T2mean	40.2 ± 5.1	34.7 ± 4.5	10.6	<.001
Middle T2max	49.7 ± 5.8	42.3 ± 5.2	11.1	<.001
Distal T2mean	41.5 ± 5.4	35.8 ± 4.6	11	<.001
Distal T2max	50.8 ± 6.0	44.0 ± 5.5	10.7	<.001

KOA = knee osteoarthritis.

### 3.3. Relationship between patellar tendon degeneration grading and T2 values

**Figure 2.**
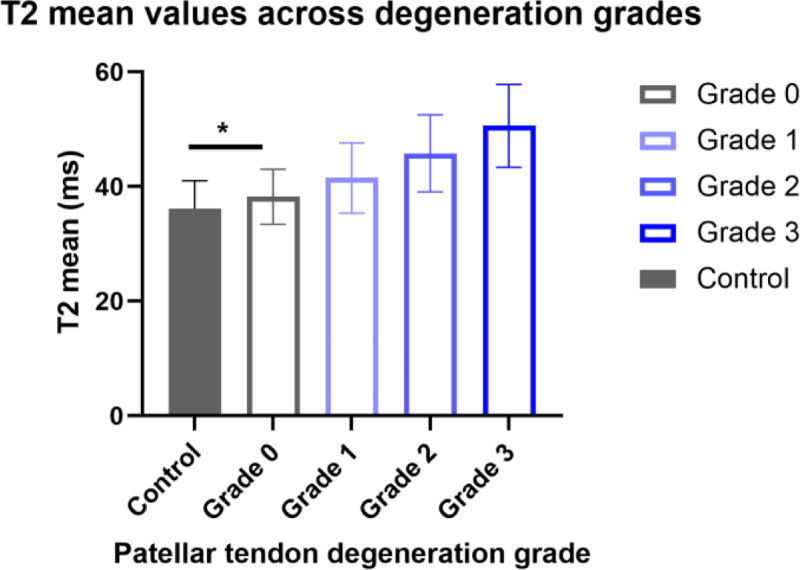
Comparison of mean T2 values across patellar tendon degeneration grades. The error bars represent the standard deviation (SD). Compared with the healthy control group, *P < .05; compared with the previous level within the KOA group, #*P* < .05 (significance marks should be indicated in the figure). Within the KOA group, the T2 value significantly increased with the increase of degeneration grade. Notably, even at grade 0, the T2 value of the KOA group was significantly higher than that of the healthy control group. KOA = knee osteoarthritis.

The measurement results of patellar tendon degeneration grading and T2 values are shown in Table 3 and Figure 2. In the healthy control group, all knees (n = 141) were classified as degeneration grade 0, with a mean T2 value of 36.1 ± 4.9 ms.

**Table 3:** Patellar tendon degeneration grade and T2mean values.

Group	Degeneration grade	Knees n (%)	T2mean (ms, Mean ± SD)
Control	Grade 0	141 (100)	36.1 ± 4.9
KOA	Grade 0	28 (9.9)	38.2 ± 4.8
	Grade 1	100 (35.2)	41.5 ± 6.1
	Grade 2	114 (40.1)	45.8 ± 6.7
	Grade 3	42 (14.8)	50.6 ± 7.2
	Total	284 (100)	–

KOA = knee osteoarthritis.

In the KOA group, 9.9% of knees (28/284) were grade 0, 35.2% (100/284) were grade 1, 40.1% (114/284) were grade 2, and 14.8% (42/284) were grade 3. With increasing degeneration grade, the mean T2 value showed a significant upward trend (*P* < .001): 38.2 ± 4.8 ms at grade 0, 41.5 ± 6.1 ms at grade 1, 45.8 ± 6.7 ms at grade 2, and 50.6 ± 7.2 ms at grade 3. Notably, the T2 values of grade 0 degeneration in the KOA group were already significantly higher than those in the healthy control group (*P* < .05).

### 3.4. Relationship between knee X-ray KL grading and patellar tendon T2 values

**Figure 3.**
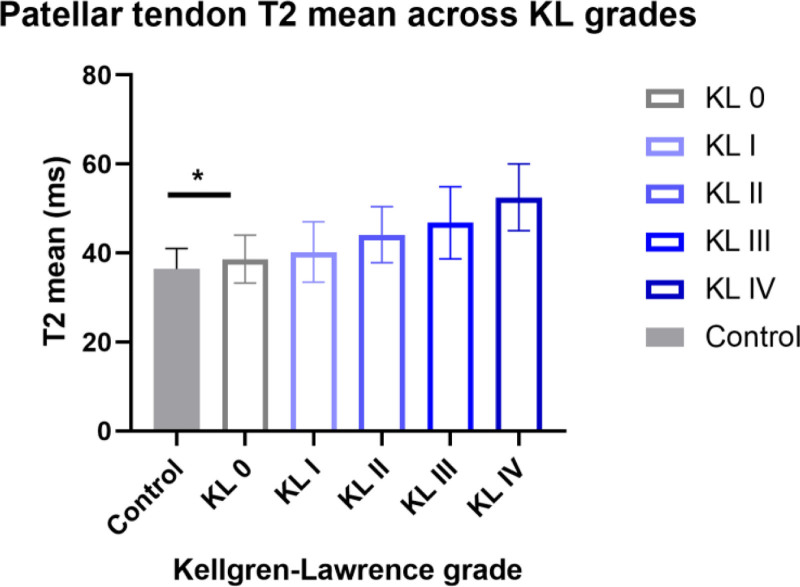
Comparison of mean T2 values of patellar tendon across different Kellgren–Lawrence grades. *Error bars represent standard deviation (SD). *P* < .05 compared with the healthy control group; #*P* < .05 compared with the previous KL grade within the KOA group. T2 values increased significantly with the progression of KL grade within the KOA group. Notably, the T2 value in KOA patients with KL grade 0 was already significantly higher than that in the healthy control group. KOA = knee osteoarthritis.

The corresponding relationship between knee X-ray KL grading and mean patellar tendon T2 values is shown in Table 4 and Figure 3. In the healthy control group, all knees (n = 141) were classified as KL grade 0, with a mean T2 value of 36.4 ± 4.6 ms.

**Table 4:** Kellgren–Lawrence (KL) grade and T2mean values.

Group	KL grade	Knees n (%)	T2mean (ms, mean ± SD)
Control	KL 0	141 (100)	36.4 ± 4.6
KOA	KL 0	15 (5.3)	38.6 ± 5.4
	KL I	55 (19.4)	40.2 ± 6.8
	KL II	113 (39.8)	44.1 ± 6.3
	KL III	72 (25.4)	46.8 ± 8.1
	KL IV	29 (10.2)	52.5 ± 7.5
	Total	284 (100)	–

KOA = knee osteoarthritis.

In the KOA group, the distribution of KL grades was as follows: 5.3% (15/284) were KL grade 0, 19.4% (55/284) were KL grade I, 39.8% (113/284) were KL grade II, 25.4% (72/284) were KL grade III, and 10.2% (29/284) were KL grade IV. Analysis showed that with increasing KL grade, the mean patellar tendon T2 value exhibited a significant upward trend (*P* < .001): 38.6 ± 5.4 ms at KL grade 0, 40.2 ± 6.8 ms at KL grade I, 44.1 ± 6.3 ms at KL grade II, 46.8 ± 8.1 ms at KL grade III, and reaching the highest value of 52.5 ± 7.5 ms at KL grade IV. Notably, KOA patients with KL grade 0 already had patellar tendon T2 values significantly higher than those in the healthy control group (*P* < .05).

### 3.5. Diagnostic performance of patellar tendon T2 mapping values for moderate-to-severe degeneration

ROC curve analysis was used to evaluate the diagnostic performance of T2 values in different patellar tendon regions for detecting moderate-to-severe degeneration (≥ grade 2). The results are shown in Table 5. The AUC of the T2mean values in the proximal, middle, and distal regions of the patellar tendon were 0.82 (95% CI: 0.77–0.86), 0.79 (95% CI: 0.74–0.84), and 0.81 (95% CI: 0.76–0.85), respectively, all demonstrating good diagnostic value.

**Table 5:** Diagnostic performance of T2 values for detecting moderate-to-severe degeneration (≥Grade 2).

Region	AUC (95% CI)	Optimal cutoff (ms)	Sensitivity	Specificity	Youden index
Proximal T2mean	0.82 (0.77–0.86)	42.5	0.76	0.75	0.51
Middle T2mean	0.79 (0.74–0.84)	40	0.72	0.73	0.45
Distal T2mean	0.81 (0.76–0.85)	41	0.74	0.76	0.5

Proximal T2mean showed the highest AUC, though differences with middle and distal segments were not statistically significant (DeLong test, *P* > .05).

AUC = area under the curve.

When 42.5 ms was used as the optimal cutoff value for proximal T2mean, the sensitivity was 76%, specificity was 75%, and the Youden index was 0.51. The optimal cutoff values for middle and distal T2mean were 40 ms and 41 ms, with sensitivities of 72% and 74% and specificities of 73% and 76%, respectively. Although the AUC value of proximal T2mean was the highest, DeLong test results showed no statistically significant difference in diagnostic performance among the 3 regions (*P* > .05).

### 3.6. Correlation and regression analysis between patellar tendon T2 values and clinical symptoms

The correlation analysis results between patellar tendon T2 values and patients’ clinical symptoms are shown in Table 6. The study found that the mean T2 values of the proximal, middle, and distal regions of the patellar tendon were all significantly positively correlated with patients’ VAS pain scores and WOMAC total scores (all *P* < .001). Among them, the proximal T2mean had the highest correlation coefficients with VAS score (*r* = 0.43) and WOMAC total score (*r* = 0.46), indicating that proximal T2 values were most closely associated with clinical symptoms.

**Table 6:** Correlation between patellar tendon T2 values and clinical symptoms.

Variable	Proximal T2mean	Middle T2mean	Distal T2mean
VAS (0–10)	0.43 (*P* < .001)	0.37 (*P* < .001)	0.40 (*P* < .001)
WOMAC total score (0–96)	0.46 (*P* < .001)	0.41 (*P* < .001)	0.44 (*P* < .001)

VAS = visual analog scale, WOMAC = Western Ontario and McMaster Universities Osteoarthritis Index.

After adjusting for potential confounding factors such as age, sex, BMI, and KL grade, multivariate linear regression analysis (Table 7) showed that the mean T2 value of the patellar tendon was an independent influencing factor of VAS scores and WOMAC total scores. For every 10 ms increase in mean T2 value, the VAS score increased by 1.1 points (95% CI: 0.80–1.50, *P* < .001), and the WOMAC total score increased by 7.6 points (95% CI: 5.1–10.1, *P* < .001). The model explained 38% of the variance in VAS scores and 42% of the variance in WOMAC total scores.

**Table 7:** Regression analysis between patellar tendon T2 values and clinical symptoms (adjusted for age, sex, BMI, KL grade).

Outcome	Predictor	β (per 10 ms ↑ in mean T2)	95% CI	*P* value	Adjusted *R*^2^
VAS	Mean tendon T2mean	1.1	0.80–1.50	<.001	0.38
WOMAC total score	Mean tendon T2mean	7.6	5.1–10.1	<.001	0.42

BMI = body mass index, KL = Kellgren-Lawrence, VAS = visual analog scale, WOMAC = Western Ontario and McMaster Universities Osteoarthritis Index.

## 4. Discussion

The results of this study showed that the T2 mapping values of the proximal, middle, and distal regions of the patellar tendon in KOA patients were significantly higher than those of healthy controls and increased progressively with higher patellar tendon degeneration grades and X-ray KL grades. This finding suggests that T2 mapping can sensitively capture microstructural alterations in the patellar tendon and that even at early stages when conventional imaging shows no obvious abnormalities, T2 values are already elevated. The underlying mechanism may involve disruption of the tendon’s collagen architecture, increased water content, and matrix degradation. Disorganization and fragmentation of collagen fibers reduce local magnetic field homogeneity, while the accumulation of free water molecules prolongs transverse relaxation time, jointly resulting in elevated T2 values. These changes reflect early biochemical and structural deterioration of the tendon and are consistent with mechanisms reported in previous T2-mapping studies of cartilage and meniscus.^[13,14]^

Further analysis revealed that the degree of patellar tendon degeneration was not only related to imaging grading but also significantly associated with patients’ pain severity and functional impairment. Multivariate regression results indicated that T2 values remained independent predictors of VAS and WOMAC scores after adjusting for confounding factors such as age, sex, BMI, and KL grade. This highlights that while T2 mapping reflects intrinsic degenerative changes of the tissue, it also captures their intrinsic connection with clinical symptoms.^[15]^ In other words, patellar tendon T2 values may serve as a bridging indicator between imaging and clinical manifestations, with potential value for disease grading and prognostic assessment. Notably, this study found that even in KOA patients with KL grade 0 or MRI degeneration grade 0, patellar tendon T2 values were significantly higher than those of healthy controls, suggesting that T2 mapping has high sensitivity in detecting early occult degeneration.^[8,11]^ This provides a potential imaging basis for early diagnosis and intervention of KOA.

ROC curve results showed that patellar tendon T2 values had good diagnostic performance for identifying moderate-to-severe degeneration, with the proximal region showing the highest AUC; however, no statistically significant differences were found compared with the middle and distal regions. This finding suggests that T2 values from different regions can all effectively distinguish between mild and moderate-to-severe degeneration, allowing flexible selection in clinical practice depending on image quality and lesion location.^[16,17]^ Combined with the correlation and regression analysis results, this study emphasizes that proximal patellar tendon T2 values had the closest relationship with clinical symptoms, which may be related to its anatomical position bearing greater tensile stress and its vascular supply characteristics, making it more prone to degeneration.^[18]^

Prior T2-mapping studies in KOA have mainly targeted cartilage and meniscus, where elevated T2 reflects collagen disruption and increased water content and can precede morphologic change.^[13–15,18]^ Our work extends this paradigm to the patellar tendon, showing that tendon T2 values increase with degeneration grade and KL grade and correlate with VAS/WOMAC, thereby linking quantitative imaging to symptoms – an aspect less emphasized in tendon-focused reports.^[14,15,18]^ Methodologically, we used a relatively large cohort and regional analysis (proximal/middle/distal); the proximal segment showed the strongest association with symptoms, suggesting spatial heterogeneity that may aid clinical assessment. These findings indicate that patellar tendon T2 mapping provides incremental value beyond cartilage/meniscus evaluations by capturing extra-articular degenerative change with direct clinical relevance.

Despite these clinically meaningful results, this study has several limitations. First, it was a single-center retrospective study. Although the sample size was relatively large, selection bias may still exist, and the findings require validation in multicenter, prospective cohorts. Second, the participants were all from the same geographic region in southern China. Differences in regional lifestyle, diet, or occupational activity, which might influence tendon properties, were not specifically analyzed because such variables are difficult to quantify and standardize in retrospective data collection. Third, T2 mapping values are susceptible to the influence of scanning parameters, coil type, and motion artifacts. Although a unified scanning protocol and strict quality control were implemented, systematic errors could not be completely avoided. Fourth, no direct comparison with histological gold standards was available, and tendon degeneration assessment relied mainly on MRI findings, which may involve some subjectivity. Finally, while T2 values were significantly associated with clinical symptoms, pain and functional impairment in KOA are multifactorial – affected by synovitis, bone marrow lesions, and periarticular soft-tissue changes – so a single tendon parameter cannot fully account for symptom origin.

In conclusion, this study demonstrates that MRI T2 mapping can sensitively reflect degenerative changes of the patellar tendon in KOA patients. T2 values increase with higher degeneration grades and KL grades and are closely associated with pain and functional impairment. T2 mapping not only enables early detection of occult degeneration but also serves as an independent quantitative predictor of clinical symptoms, providing important value for imaging evaluation and clinical management of KOA. Future multicenter, large-sample, prospective studies are needed to further validate its clinical applicability, and comprehensive evaluation should be conducted in combination with other advanced imaging biomarkers (such as T1ρ and UTE), thereby promoting early diagnosis and precise intervention of KOA.

## Author contributions

**Conceptualization:** Kaifu Wang, Cuixia Mao, Xiaoyi Lao, Zhanqing Zhang, Xing Li, Xiesheng Xu.

**Data curation:** Kaifu Wang, Jie Men, Xiaoyi Lao, Xiesheng Xu.

**Formal analysis:** Kaifu Wang, Jie Men, Cuixia Mao, Zhanqing Zhang, Xing Li, Xiesheng Xu.

**Funding acquisition:** Xiaoyi Lao, Zhanqing Zhang, Xing Li, Xiesheng Xu.

**Investigation:** Jie Men, Xiesheng Xu.

**Writing – original draft:** Jie Men, Cuixia Mao, Zhanqing Zhang, Zuanming Huang, Xiesheng Xu.

**Writing – review & editing:** Cuixia Mao, Zhanqing Zhang, Zuanming Huang, Xiesheng Xu.

## References

[1] Chen D Shen J Zhao W. Osteoarthritis: toward a comprehensive understanding of pathological mechanism. Bone Res. 2017;5:16044. 28149655 10.1038/boneres.2016.44 PMC5240031

[2] Tong L Yu H Huang X. Current understanding of osteoarthritis pathogenesis and relevant new approaches. Bone Res. 2022;10:60. 36127328 10.1038/s41413-022-00226-9 PMC9489702

[3] Zhang Y Jordan JM. Epidemiology of osteoarthritis. Clin Geriatr Med. 2010;26:355–69. 20699159 10.1016/j.cger.2010.03.001 PMC2920533

[4] Giorgino R Albano D Fusco S Peretti GM Mangiavini L Messina C. Knee osteoarthritis: epidemiology, pathogenesis, and mesenchymal stem cells: what else is new? An update. Int J Mol Sci. 2023;24:6405. 37047377 10.3390/ijms24076405 PMC10094836

[5] Kohn MD Sassoon AA Fernando ND. Classifications in brief: Kellgren–Lawrence classification of osteoarthritis. Clin Orthop Relat Res. 2016;474:1886–93. 26872913 10.1007/s11999-016-4732-4 PMC4925407

[6] Benjamin M Kaiser E Milz S. Structure–function relationships in tendons: a review. J Anat. 2008;212:211–28. 18304204 10.1111/j.1469-7580.2008.00864.x PMC2408985

[7] Dickson DM Theis JC Burt E. An evaluation of quadriceps and patellar tendon elasticity in knee osteoarthritis. Sci Rep. 2022;12:17064.36257969

[8] Breda SJ Schmidt TA Klose-Jensen R. Tissue-specific T2* biomarkers in patellar tendinopathy by subregional quantification using 3D ultrashort echo time MRI. J Magn Reson Imaging. 2020;52:801–10. 10.1002/jmri.27108 PMC7496783 32108398

[9] O’Neill TW Felson DT. Mechanisms of osteoarthritis (OA) pain. Curr Osteoporos Rep. 2018;16:611–6. 30155845 10.1007/s11914-018-0477-1 PMC6153568

[10] Eijgenraam SM Thomas KE Nardo L. T2 mapping and structural imaging of the knee using a single 5-min quantitative double-echo steady-state (qDESS) scan. Eur Radiol. 2019;29:6257–68.

[11] Khandelwal R Tiwari R Rai N. High-resolution T2* mapping in assessment of knee articular cartilage on 3T MRI. Pol J Radiol. 2022;87:e77–87. 10.1016/j.jcot.2022.101823 PMC8894233 35251934

[12] Bellisari FC Pitino R Lovreglio R. T2-mapping MRI evaluation of patellofemoral cartilage in patients submitted to intra-articular platelet-rich plasma injections. Radiol Med. 2021;126:1307–17. 10.1007/s11547-021-01372-6 PMC8292236 34008045

[13] Maizlin ZV Lutz AM Schweitzer ME. T2 mapping of articular cartilage of glenohumeral joint: feasibility in routine clinical imaging. J Magn Reson Imaging. 2009;30:678–83.19711418

[14] Pownder SL Hayashi K Lin BQ. Differences in the magnetic resonance imaging parameter T2* may be identified during the course of canine patellar tendon healing: a pilot study. Quant Imaging Med Surg. 2021;11:1234–46. 33816163 10.21037/qims-20-684 PMC7930665

[15] Zhao H Li H Liang S Wang X Yang F. T2 mapping for knee cartilage degeneration in young patients with mild symptoms. BMC Med Imaging. 2022;22:72. 35436880 10.1186/s12880-022-00799-1 PMC9017029

[16] Dautry R Bousson V Manelfe J. Correlation of MRI T2 mapping sequence with knee pain location in young patients with normal standard MRI at 3.0 Tesla. J Belg Soc Radiol. 2014;97:11–6. 10.5334/jbr-btr.364 24765764

[17] Verschueren J Wouters K Molken MP. T2 mapping of healthy knee cartilage: multicenter multivendor reproducibility and variation. Quant Imaging Med Surg. 2021;11:1513–24. 10.21037/qims-20-674 PMC7930682 33816164

[18] Baum T Joseph GB Carballido-Gil D. Cartilage and meniscal T2 relaxation time as non-invasive biomarkers for the progression of knee osteoarthritis: data from the osteoarthritis initiative. Osteoarthritis Cartilage. 2013;21:1474–82. 23896316 10.1016/j.joca.2013.07.012 PMC3929642

